# The regulation of totipotency transcription: Perspective from *in vitro* and *in vivo* totipotency

**DOI:** 10.3389/fcell.2022.1024093

**Published:** 2022-10-26

**Authors:** Haiyan Xu, Hongqing Liang

**Affiliations:** Division of Human Reproduction and Developmental Genetics, Women’s Hospital, and Institute of Genetics, Zhejiang University School of Medicine, Hangzhou, China

**Keywords:** embryonic stem cell, totipotency, transcription regulation, epigenetic regulation, developmental potential

## Abstract

Totipotency represents the highest developmental potency. By definition, totipotent stem cells are capable of giving rise to all embryonic and extraembryonic cell types. In mammalian embryos, totipotency occurs around the zygotic genome activation period, which is around the 2-cell stage in mouse embryo or the 4-to 8-cell stage in human embryo. Currently, with the development of *in vitro* totipotent-like models and the advances in small-scale genomic methods, an in-depth mechanistic understanding of the totipotency state and regulation was enabled. In this review, we explored and summarized the current views about totipotency from various angles, including genetic and epigenetic aspects. This will hopefully formulate a panoramic view of totipotency from the available research works until now. It can also help delineate the scaffold and formulate new hypotheses on totipotency for future research works.

## 1 Introduction

The very first demonstration of totipotent cells in the embryo was from the works of Tarkowski, in which a single blastomere isolated from a 2-cell (2C) stage embryo was proven to be able to generate a whole adult mouse (Tarkowski, 1959). By definition, totipotency refers to the developmental potential of cells capable of giving rise to the whole fertile organism (Redo and Brickman, 2020; Malik and Wang, 2022). Sometimes, functional assays, which revealed the capacity of stem cells being able to differentiate into all embryonic and extraembryonic lineages, are also recognized as a demonstration of totipotency. Apart from the definition in the perspective of developmental potentials, molecular criteria, including transcriptomic and epigenetic features, are also used to assess the totipotency state (Malik and Wang, 2022). In mouse embryos, totipotency emerges from the zygote to the 2-cell embryonic stage (Lu and Zhang, 2015). In addition, embryonic cells up to the 4-cell stage possess differentiation capacity toward all embryonic and extraembryonic lineages, thus also recognized as totipotent. Following these stages, the embryonic cells start to commit to fate segregation, first toward inner cell mass (ICM), which gives rise to fetal tissues, and trophectoderm (TE), which gives rise to extraembryonic placental tissues.

The transcription network regulating totipotency undergoes dynamic wiring from 1C to 4C stages and expands the zygotic genome activation (ZGA) period. Shortly after fertilization, maternal RNAs and proteins stored in the oocyte start to be degraded, a process known as maternal decay; at the same time, the zygotic genome begins to be transcribed, known as ZGA (Sha et al., 2019). ZGA usually occurs in two subsequent steps: a minor ZGA which predominantly takes place in the paternal pronucleus and a more substantial major ZGA which occurs in a 2-cell mouse embryo or 8-cell human embryo (Eckersley-Maslin et al., 2019). Minor ZGA is a prerequisite of major ZGA. Transient inhibition of minor ZGA with transcription inhibitors resulted in embryonic arrest at the 2-cell stage and major ZGA defect (Abe et al., 2018). How ZGA is triggered and how it contributes to the wiring of the totipotency transcription network and developmental potential are essential questions in the early embryogenesis field. So far, various biological pathways, such as transcription, cell cycle, nuclear/cytoplasmic ratio, or translation of key ZGA transcription factors (TFs), have been implicated in ZGA initiation and are also found to be important for totipotency establishment (Jukam et al., 2017).

Yet, comprehensive studies about totipotency and ZGA regulators have been limited due to the availability of cells in early embryos. However, identification and establishment of *in vitro* totipotent-like stem cell culture systems have significantly enabled the dissection of the totipotency regulation. Currently, different *in vitro* totipotency models share similarities but also bear substantial differences compared to *in vivo* totipotency states. Under serum/leukemia inhibitory factor (LIF) culture conditions, around 1%–2% of mouse embryonic stem cells (mESCs) were found to exhibit transcription features such as the 2C embryos and were known as 2C-like embryonic stem cells (2CLCs) (Macfarlan et al., 2012; Li and Izpisua Belmonte, 2018). 2CLCs possess the ability to contribute to both embryonic and extraembryonic tissues, although the functionality of differentiated cells remains to be evaluated (Macfarlan et al., 2012). The somatic cell nuclear transfer (SCNT) technique is also another way of reprogramming to access totipotent stem cells (Gurdon, 1962). In this context, 2CLCs have higher efficiencies of SCNT due to their similarity to 2C embryos (Ishiuchi et al., 2015). Since the identification of 2CLCs in 2012, a large number of the molecular mechanisms regulating the totipotency state have been revealed using this *in vitro* totipotency model. Recently, human 8-cell-like cells (8CLCs) were also discovered to exist in the heterogeneous human naïve embryonic stem cells, which transcriptionally resemble the 8-cell human embryos (Mazid et al., 2022; Taubenschmid-Stowers et al., 2022). In addition, small molecules targeting different signaling pathways have been used to generate different totipotent-like models *in vitro*. Expanded potential stem cells (EPSCs) were first established by Liu and Deng laboratories (Yang J. et al., 2017; Yang Y. et al., 2017), which can contribute to both embryo proper and trophectoderm lineages in chimera assays (Yang J. et al., 2017; Yang Y. et al., 2017). Transcriptionally, EPSCs derived correlated with E4.5 to E5.5 epiblast or EpiSCs (Posfai et al., 2021). Recently, using a splicing inhibitor pladienolide B (PlaB), the totipotent blastomere-like cell (TBLC) model was established (Shen et al., 2021; Taubenschmid-Stowers et al., 2022). TBLCs show epigenetic and transcriptomic profiles similar to those of 2C–4C embryos but distinct from those of 2CLCs. It has robust embryonic and extraembryonic differentiation potential. Similarly, totipotent-like stem cell (TLSC) models were established from mESCs using small-molecule induction, in which the transcriptional and epigenetic states were found to be closer to those of 2C embryos (Yang et al., 2022). However, both TBLCs and TLSCs differentiated poorly toward some of PrE- and TE-derived lineages (Shen et al., 2021; Yang et al., 2022), questioning the developmental potential of these *in vivo* totipotent cells. Recently, the Ding laboratory recently generated a culture condition to reprogram cells to chemically induce totipotent stem cells (ciTotiSCs), resembling mouse totipotent 2C embryo stage cells at transcriptional, epigenetic, and metabolic levels (Hu et al., 2022). Excitingly, the culture conditions in the Deng laboratory can derive totipotent potential stem (TPS) cells from 2C embryos. *In vivo* chimera formation assays show that these cells have embryonic and extraembryonic developmental potentials at the single-cell level and can induce blastocyst-like structures from TPS cells *in vitro* (Xu Y. et al., 2022). In general, all these *in vitro* totipotent-like models possess chimera formation, transcriptional, and epigenetic features similar to those of totipotent embryonic cells. However, more robust functional demonstration of their developmental totipotency remains to be assessed.

Here, we explored and summarized the current understanding on regulation of totipotent stem cells and totipotency in early embryos from various genetic and epigenetic perspectives, such as transcriptional regulation, epigenetic regulation, cell cycle, DNA damage, and RNA metabolism. From the current emerging evidence, the panoramic regulation of the totipotency state will surface more.

## 2 Transcriptional regulation of the totipotency state

Specific cell states are shaped by various pathways, and among all, gene expression plays essential roles. The emergence of totipotency is accompanied by the transcription network sustaining ZGA. Both cis- and trans-genomic elements and coding and non-coding genes are actively engaged in ZGA and the establishment of the totipotent state.

### 2.1 Transcription factors in regulation of the totipotency state

Transcription factors work by recognizing specific DNA sequences and recruiting RNA polymerase for specific gene transcription. Totipotent stem cells have their own unique transcriptional regulatory network. Currently, the understanding of the totipotent transcriptional regulatory network is just beginning. A few positive and negative transcription regulators have been identified from the *in vitro* and *in vivo* totipotency models.

Among all totipotency-related TFs, *Dux*, a double homeodomain transcription factor, was the most significantly studied, which was initially found to reprogram pluripotent mESCs toward a 2C-like state (De Iaco et al., 2017; Hendrickson et al., 2017; Whiddon et al., 2017). Upon *Dux* induction, 2C-related genes including the *Zscan4* family, *Zfp352*, and 2C-specific transposable elements (TEs) were transcriptionally activated. The chromatin landscape was also reorganized to resemble that of mouse 2C embryos (Hendrickson et al., 2017). Similarly, over-expression of the *Dux* homolog *DUX4* in human ESCs can also push a totipotent-like state (De Iaco et al., 2017; Hendrickson et al., 2017; Sugie et al., 2020; Taubenschmid-Stowers et al., 2022). So far, in the 2CLC model, various transcription regulation mechanisms uncovered are centered around *Dux*. However, despite the fact that *Dux* is at the central node of the 2CLC state, knocking out *Dux* cluster genes in mouse embryos led to no dramatic developmental defect, and the mouse can survive to adulthood. Correspondingly, the transcriptome of *Dux* knock-out embryos had minimal defects around ZGA (Chen and Zhang, 2019); ZGA can still occur normally despite a slight delay (Guo et al., 2019). This implies that *Dux* plays a supportive rather than an essential role in the activation of the ZGA transcription network in early embryonic development, and parallel or redundant mechanisms exist to initiate ZGA *in vivo* to sustain the totipotent transcription network (Chen and Zhang, 2019; Guo et al., 2019; De Iaco et al., 2020; Bosnakovski et al., 2021).

Recently, a multi-copy mouse homeobox gene, *Obox4*, was identified, which potentially acted in parallel with *Dux* to initiate the ZGA network. Single knockdown of *Obox4* or *Dux* was tolerated by embryogenesis, whereas *Obox4/Dux* double knockdown completely abolished embryonic development after 2C (Guo et al., 2022). *Dux* was rapidly degraded at the end of the 2C embryo stage, which is thought to be necessary for exit from the totipotency state both *in vivo* and *in vitro*. Prolonged expression of *Dux* in 2C/4C embryos enhanced the expression of ZGA genes and TEs and led to sustenance of the 2C transcription network (Percharde et al., 2018; Guo et al., 2019). Upon ectopic *Dux* expression, embryos stalled development and eventually died after one more division (Percharde et al., 2018; Guo et al., 2019; Ruebel et al., 2019). Moreover, exogenously injected *Dux* RNAs were rapidly degraded in embryos (Guo et al., 2019). *In vitro*, prolonging *Dux* over-expression in mESCs not only resulted in increased 2CLCs but also DNA damage and apoptosis (Olbrich et al., 2021). Thus, it is possible that, for proper early embryonic development, precise quantitative and temporal regulation of *Dux* is crucial.

Maternal RNAs and proteins are transmitted to the zygote, which are important upstream mediators of ZGA. The maternal factor is required to activate *Dux* (Hendrickson et al., 2017). Paralog maternal factors *Dppa2* and *Dppa4* were identified to bind the promoters of *Dux* and activated *Dux* expression (De Iaco et al., 2019; Yan et al., 2019). In addition, another maternal factor, the pause release factor *Nelfa*, can also activate *Dux* through its interaction with DNA topoisomerase 2a (*Top2a*) (Hu et al., 2020). On the other hand, *Dux* expression reversely activated *Nelfa* (Grow et al., 2021). In addition to maternal factors, P53 and DNA-damage response (DDR) pathways were also reported to activate *Dux* and promote totipotency transcription. These pathways were also reported to be active in mouse early embryos (Atashpaz et al., 2020; Grow et al., 2021). Recently, it was reported that nuclear receptor *Nr5a2* was a crucial pioneer activator of major ZGA by promoting chromatin accessibility and was necessary for progression beyond the 2C stage (Gassler et al., 2022). Thus, multi-mechanisms may redundantly ensure the precise timing and regulation of ZGA and totipotency establishment.

*Zscan4* family members are also highly expressed transcription factors in mouse and human totipotent cells, among which *Zscan4d* is the most highly transcribed in 2C embryos, and *Zscan4c* is a major transcript in mESCs (Falco et al., 2007). *Zscan4c* interacted with 2C-specific transposable element MT2 (LTR of MERVL) through the SCAN domain, which interacted with the GBAF chromatin remodeling complex to promote the enhancer activity of MT2 (Zhang W. et al., 2019). Reduction in *Zscan4* transcript levels slowed down the transition from 2C–4C embryos (Falco et al., 2007). The *Zscan4* family is downstream of *Dux*. Interestingly, *Dppa2* and *Dppa4*, the activators of *Dux*, can also be activated by *Zscan4*. In contrast to *Dux*, *Zscan4c* cannot induce 2CLCs in *Dppa2/4* double-knockout cells (Eckersley-Maslin et al., 2019). The evidence reflects a complex transcription feedback loop between *Zscan4*, *Dppa2/4*, *and Dux in vitro* and *in vivo* totipotency states (Zhang W. et al., 2019).

In addition to activators, repressors of 2C states were also identified, many of which are active in the pluripotent state of mESCs. *Ythdc1*, an m6A reader, is required for maintenance of pluripotent mESCs, while the deletion of *Ythdc1* facilitated the reversion of mESCs to the 2C-like state through activation of *Dux* (Liu et al., 2021). Totipotency repressor, zinc finger protein *Zfp281*, can prominently inhibit *Dux*-activated transcripts and prevent mESCs from transiting back to the 2C-like state (Wen et al., 2022).

### 2.2 Transposable elements and the regulation of totipotency

**FIGURE 1:**
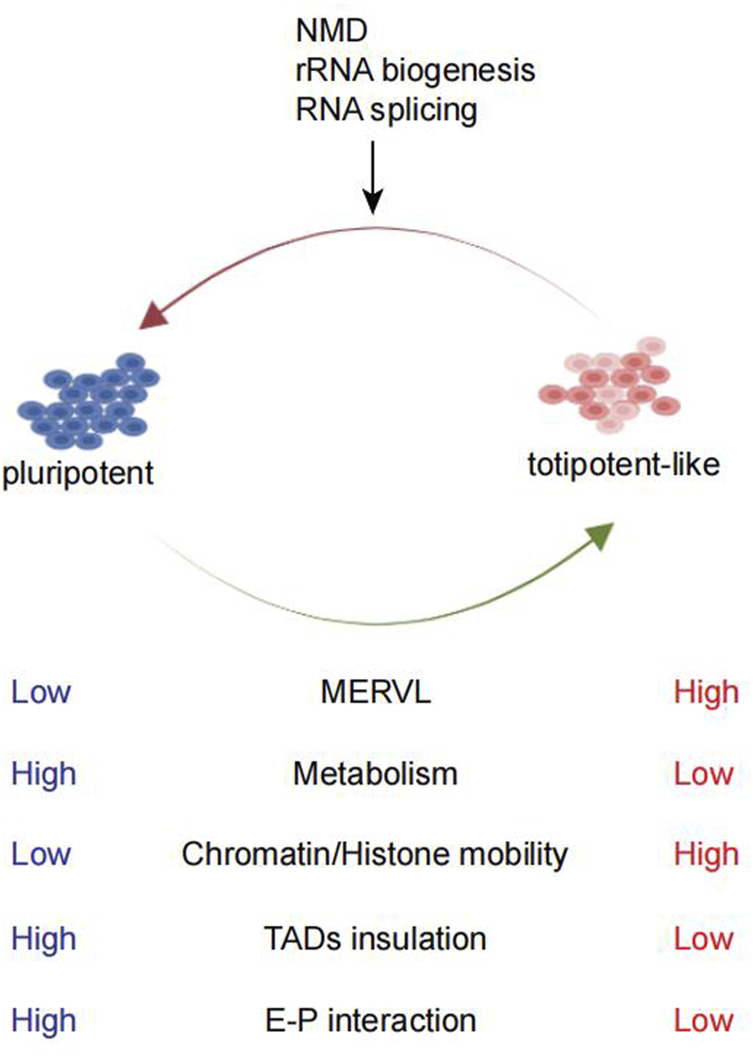
Comparison between pluripotent and totipotent-like cells and the pathways studied for totipotent-like exit. mESCs can fluctuate in and out of a transient totipotent-like state and exhibit similarities to totipotent 2C embryos. Totipotent-like cells exhibit high MERVL expression, low metabolism, high chromatin/histone mobility, weakening of TAD insulation, and E–P interaction. NMD, rRNA biogenesis, and RNA splicing are shown to promote totipotent-like exit.

TEs have the ability to transposit in the genome and constitute around 30–50% of mammalian genomes (Elbarbary et al., 2016; Fueyo et al., 2022). Mammalian totipotency has been linked with an extensive transposon-based regulatory mechanism. TEs consist of DNA and RNA transposons (or retrotransposons) (Slotkin and Martienssen, 2007; Kunarso et al., 2010). The retrotransposon was further classified into short interspersed nuclear elements (SINEs), long interspersed nuclear elements (LINEs), and endogenous retroviruses (ERVs). MERVL, the mouse ERVL sub-families, is specifically upregulated in the 2C embryos (Figure 1). Similarly, HERVL expression is also enriched in the human 8C stage (Göke et al., 2015). Both MERVL and HERVL can be bound by DUX or DUX4 (Hendrickson et al., 2017). Efficient and precise activation of MERVL plays a crucial role in early embryogenesis. Loss of MERVL expression impaired developmental progression (Huang et al., 2017; Sakashita et al., 2022), while ectopic activation of MERVL loci in mESCs switched on the 2C-related gene expression (Yang et al., 2020). Many 2C-specific ZGA genes rely on the LTR of MERVL as a promoter or enhancer (Macfarlan et al., 2011, 2012). In addition, the transcribed RNAs from MERVL can also form chimeric transcripts with coding genes to promote the totipotency state (Peaston et al., 2004; Macfarlan et al., 2012). In addition to MERVL, LINE-1 element transcription is observed most in a 2C embryo (Jachowicz et al., 2017), which can function as a nuclear RNA scaffold to recruit the KAP1/nucleolin (NCL) complex to repress *Dux* in mESCs and pre-implantation mouse embryos (Percharde et al., 2018). In early human embryos, hominoid-specific retrotransposon SINE-VNTR-Alu (SVA) elements are activated by H3K9me3 reprogramming, leading to activation of major ZGA gene expression (Xu R. et al., 2022; Yu et al., 2022).

Despite the emerging evidence of TEs’ function in ZGA and totipotency regulation, how their specific activation and repression are coupled to the totipotent transcription network remains poorly understood. ERVs bear motifs to recruit transcription factors or epigenetic modifiers. In the case of MERVL, it can be bound and activated by maternal factor *Stella* (Huang et al., 2017). Long non-coding RNAs and small RNAs involved in totipotency also participate in regulation of MERVL (Svoboda, 2017), such as *miR-34a* and *miR-344*. *miR-34a* has been demonstrated to be important for activating MERVL and promoting the 2C totipotency state through activating *Gata2* expression (Choi et al., 2017). Meanwhile, *miR-344* activated by *Dux* can post-transcriptionally repress ZMYM2-LSD1, a transcriptional corepressor complex, leading to MERVL activation (Yang et al., 2020). Totipotency-related TEs can also be regulated by various epigenetic factors, such as histone H3 variants (Elsässer et al., 2015), H3K9 methyltransferases (Rowe et al., 2010; Maksakova et al., 2013), and histone chaperones (Hatanaka et al., 2015; Ishiuchi et al., 2015; Yang et al., 2015). The facilitates chromatin transcription (FACT) complex is a histone H2A/H2B chaperone, which can repress MERVL and ERV-chimeric transcripts (Chen et al., 2020) through regulating the expression of *Nsd2* (Gao et al., 2021). YTHDC1, the m6A reader, can bind and direct ERVK, intracisternal A particles, (IAPs) and LINE1 RNAs to their loci, which led to silencing of these retrotransposons through deposition of histone H3K9me3, facilitating the reprogramming toward totipotent-like states (Liu et al., 2021). In addition, the zinc finger protein, ZFP809, can bind to MERVL through the zinc finger domain and silence MERVL through its KRAB domains by recruiting TRIM28, NURD (histone deacetylase), and SETDB1 (histone methyltransferase) (Wolf et al., 2015; Wang and Goff, 2017).

In conclusion, although numerous regulating factors have been studied so far that can promote the emergence of totipotent-like cells which acquire a generally similar chromatin landscape and epigenetic modifications as 2C embryos, only a few genes acquire the epigenetic features of 2C embryos (Zhang Y. et al., 2021), and further epigenetic regulation needs to be revealed.

## 3 Epigenetic regulation of totipotency

Totipotency is associated with globally less repressive histone marks and increased chromatin accessibility (Ishiuchi et al., 2015; Eckersley-Maslin et al., 2016). On the other hand, during the transition toward pluripotency, decreased histone mobility can be observed (Bošković et al., 2014) (Figure 1). Recently, further remodeling of pericentromeric heterochromatin and reconstruction of a pluripotency-specific broad H3K4me3 domain facilitated more efficient reprogramming of pluripotent ESCs toward TLSCs, which bears more epigenetic resemblance to 2C embryos (Yang et al., 2022). This suggests that epigenetic reprogramming endues the essential features for the totipotency state. Currently, given that only a few genes acquire 2C-embryo epigenetic signatures (Zhang Y. et al., 2021), further efforts to reprogram the epigenetic modification toward the 2C embryo state remain a major challenge.

### 3.1 Regulation of DNA methylation in the totipotency state

Global DNA demethylation occurs soon after fertilization until the blastocyst stage, while increasing methylation was observed during differentiation (Smith et al., 2012). Wolf Reik group, identified a global DNA methylation dynamic during the reprogramming of ESCs toward totipotent-like cells (Eckersley-Maslin et al., 2016). Loss of DNMTs led to the reprogramming into the MERVL^+^Zscan4^+^ totipotent-like state which was triggered by the genome-wide DNA demethylation. In contrast, during the exit from the totipotent state, the restoration of DNMT protein levels led to establishment of genome-wide DNA methylation (Eckersley-Maslin et al., 2016). DNA demethylation allows the activation of transcription factors, leading to the up-regulation of the MERVL and chromatin decompaction. Furthermore, the DNA demethylase TET may act as an epigenetic barrier for the transition from pluripotent to intermediate and 2C-like states by facilitating KAP1 recruitment (Lu et al., 2014). Correspondingly, *Tet*1/2/3 triple knock-out cells (TKOs) were more poised to transition to a 2C-like state (Lu et al., 2014; Qiu et al., 2020). In apparent contradiction to these findings, *Gadd45*, the regulator of TET-mediated DNA demethylation, promoted 2C-like state transition. *Gadd45a/b/g* TKOs exhibited DNA hypermethylation with impaired transition to a 2C-like state. *Gadd45a/b* double knock-out mouse embryos showed embryonic lethality, with abrogated ZGA and developmental arrest (Schüle et al., 2019). A negative regulator of *Tet*, *Smchd1*, which is involved in X chromosome inactivation in female cells (Blewitt et al., 2008), can also inhibit the 2C transcription program (Huang et al., 2021). In general, *Tet* may play a dual role in the regulation of the transition toward the 2C stage: first, repression of LINE1 through the KAP1/TRIM28/NCL complex led to silencing of *Dux* (Lu et al., 2014; Percharde et al., 2018; Schüle et al., 2019). Second, *Gadd45* and *Smchd1* targeted *Tets* to promote the 2C conversion *via* demethylation and protection against *de novo* methylation (Schüle et al., 2019).

In conclusion, totipotent embryos and totipotent-like cells are both hypomethylated; however, blastocyst and naïve pluripotent stem cells also possess the hypomethylated genome (Leitch et al., 2013). Therefore, hypomethylation could be a general epigenetic feature of early embryo development but not specific for totipotency regulation.

### 3.2 Regulation of histone modification in the totipotency state

Histone modifications play important roles in many biological processes by regulating chromatin states. Appropriate remodeling of histone modification is critical for embryonic development and totipotency reprogramming (Yang et al., 2021). Treatment with histone deacetylase (HDAC) inhibitors on mESCs can result in the activation of MERVL and/or *Zscan4*, with the emergence of 2CLCs (Dan et al., 2015; Walter et al., 2016), while knocking down the chromatin remodeler *Chd5* (Hayashi et al., 2016), Hnrnpk (Thompson et al., 2015), or members in the repressive chromatin complexes such as *Kap-1*, *Kdm1a*, *G9a*, *Hp1*, or *Rybp* similarly led to 2C-like transition (Ishiuchi and Torres-Padilla, 2013).

Core histones H2A, H3.1, and H3.2 are expressed at a high level in 2C embryos but gradually declined as embryos develop (Tian et al., 2020). *Caf-1* is responsible for deposition of histones H3 and H4 during DNA synthesis, and depletion of the p150 subunit of CAF-1 leads to chromocenter loss in pluripotent ESCs and transition into 2CLCs (Houlard et al., 2006; Ishiuchi et al., 2015). In addition, H3.3 was directly associated with *Dux* loci and inhibited *Dux* expression (Tian et al., 2020). H3.3 is also enriched around MERVL in 2CLCs and 2C embryos, coincident with the onset of MERVL expression (Nakatani et al., 2022). Induction of 2CLCs was found following p150 depletion (Yang et al., 2022), supporting that *Caf-1* depletion-induced 2C-like states was associated with altered histone deposition.

It was found that incomplete depletion of H3K9me3 was a key reason underlying the arrest of somatic cloned embryos at the ZGA stage (Matoba et al., 2014; Liu et al., 2016), and abnormal enrichment of H3K9me3 also severely hindered pre-implantation embryonic development in mice (Burton et al., 2020). The correct establishment of H3K9me3 modifications on ERVs was recently found to be depending on *Dux* (Xu R. et al., 2022). At the human 8C stage, H3K9me3 was reprogrammed on homology-specific retrotransposons of SVA (SINE-VNTR-Alu elements), remodeling the enhancer function of SVA to promote totipotent gene expression (Yu et al., 2022). *Setdb1*, a histone H3K9 methyltransferase, is essential for embryonic development. *Setdb1* deficiency initiated 2C-like transition in a *Dux*-dependent manner (Wu et al., 2020). *Kdm5b*, an H3K4 demethylase, can recruit SETDB1 to repress ERVs such as MMVL30 (Zhang S.-M. et al., 2021). Inhibition of *Kdm5b* promoted the remodeling of broad H3K4me3 regions, which facilitated the stabilization of totipotency transcription (Yang et al., 2022). Aberrant acetylated regions were epigenetic barriers for efficient totipotency reprogramming in SCNT, and histone deacetylase inhibitor TSA can improve the efficiency of *Dux* cluster gene activation in SCNT (Yang et al., 2021). Globally, MERVL^+^ cells have increased levels of histone acetylation (Macfarlan et al., 2012; Ishiuchi et al., 2015). Similarly, Zscan4^*+*^ cells showed increase in H3K27ac modification in both totipotent genes and retrotransposons (Akiyama et al., 2015).

In mouse 2C embryos, chromatins are associated with a broad decrease in H3K27me3, H3K36me3 and H3K9me3 after fertilization, while a broad increase in H3K27ac modification in the 2C stage is observed (Fu et al., 2020b). A unique chromatin domain structure relating to H3K27me3 is maintained up to the 8C stage and diminishes thereafter (Zheng et al., 2016; Collombet et al., 2020). Most of the differentially expressed genes in 2CLCs displayed globally decreased H3K4me3 and H3K27me3 levels, while *de novo* H3K4me3 deposition in the promoter regions was found in highly upregulated 2C genes (Zhang Y. et al., 2021). Augmented H3K27ac deposition on MT2 was also observed (Zhang W. et al., 2019). During early human embryogenesis, wider promoter H3K4me3 marks were easily observable at the 4C stage, and a subset of them remained at the 8C stage. However, compared to promoter H3K4me3, the weaker but widespread distal H3K4me3 mark decreased at the 8C stage. Also, there are no H3K27me3 signals in human embryos at the ZGA stage (Xia et al., 2019; Xu et al., 2021). Overall, in the perspective of histone modifications, *in vitro* totipotent-like cells displayed some similarities toward totipotent embryos, including broad decreased levels of H3K27me3 and H3K4me3 and an increased level of H3K27ac (Akiyama et al., 2015; Zhang Y. et al., 2021).

SUMOylation is largely observed on histones and potentially modulates chromatin structure and transcription (Cubeñas-Potts and Matunis, 2013). The experiments disrupting SUMOylation in embryos revealed its importance on mammalian development (Nacerddine et al., 2005; Lomelí and Vázquez, 2011). SUMOylation can act on chromatin to maintain cellular identity. For example, loss of SUMOylation in ESCs leads to a genome-wide decline of H3K9me3-dependent heterochromatin and converts ESCs into a 2C-like state by releasing PRC1.6 and KAP1/SETDB1 complexes from the *Dux* loci (Cossec et al., 2018; Theurillat et al., 2020). In addition, histone H1, which is crucial for maintaining heterochromatin, was controlled by SUMO2/3. This modification fixated H1 on the heterochromatin in ESCs and prevented the expression of 2C genes. Depletion of SUMOylation resulted in chromatin condensation and H1 expulsion. As a result, the 2C transcriptional program was activated with an increased H3K27ac level (Sheban et al., 2022). The DPPA2 protein can also be SUMOylated (Theurillat et al., 2020), and this was negatively regulated by SUMO E3 ligase PIAS4. Depleting *Pias4* is sufficient to activate *Dppa2* and the 2C-like transcription program (Yan et al., 2019). Although SUMOylation is important during mammalian development and 2CLC reprogramming, its function in *in vivo* totipotent embryos still awaits further elucidation.

### 3.3 Metabolic influence of totipotency epigenetics

The metabolic state of a cell is a key feature of cellular identity and has been linked to cellular plasticity. During reprogramming to 2CLCs, the metabolic state of cells changed. 2C embryos and 2CLCs exhibited substantial distinctions compared to pluripotent mESCs (Zhao et al., 2021) and generally displayed a low metabolism (Figure 1) level with decreased glycolysis and respiratory chain activity and lower reactive oxygen species (ROS) levels but increased glucose uptake (Rodriguez-Terrones et al., 2020). A screening revealed metabolites such as sodium l-lactate, d-ribose, and sodium acetate as 2CLC inducers (Rodriguez-Terrones et al., 2020). Likewise, related studies have found that chemical inhibition of glycolysis was sufficient to promote fate conversion toward 2CLC (Hu et al., 2020). The underlying influences of metabolism on cell fate decision largely depended on the effect of metabolites in epigenome remodeling (Britt et al., 2020; Zhao et al., 2021), such as the reduction in l-2-hydroxyglutarate was required for embryonic early histone demethylation (Zhao et al., 2021). However, with PCA analysis, 2CLCs were extremely different from 2C embryos and 2CLCs were very close to ESCs (Zhao et al., 2021). Perhaps, we could establish a unique totipotent stem cell model which is closer to the 2C embryo by altering the metabolome of ESCs.

### 3.4 Regulation of the 3D genome in the totipotency state

In mammalian cells, three-dimensional (3D) genome is critical for gene regulation and is highly dynamic during embryo development. The DNA in the nucleus is hierarchically organized into chromosome territories, compartments, TADs, and chromatin loops. In general, dramatic reprogramming of the 3D chromatin architecture occurs after fertilization. Also, substantial chromatin compaction occurs during the 1-cell to 2-cell transition, resulting in the formation of large chromatin blocks that accumulate near the nuclear envelope (Zheng and Xie, 2019). A higher-order chromatin structure and TADs start to be established from the late 2C stage in mouse embryos (Ke et al., 2017; Fu et al., 2020b), while during human embryonic development, TAD structure is gradually established after fertilization. Notably, blocking ZGA can inhibit TAD establishment in human embryos but not in mice (Chen et al., 2019).

Previous studies revealed both 2CLCs and 2C embryos display more relaxed chromatin structure than mESCs, including an overall weakened enhancer–promoter (E–P) interactions and TAD insulation (Ishiuchi et al., 2015; Zhu et al., 2021b) (Figure 1). While the weakened E–P interaction was associated with mESC enhancer inactivation and down-regulation of pluripotency genes, the weakened TAD insulation may promote contacts between the putative new 2C enhancers and neighboring 2C genes (Zhu et al., 2021b). Typically, chromocenters and major satellite transcription are features of the totipotency state (Houlard et al., 2006). However, whether the change in 3D chromatin structures is a cause or consequence of the totipotency state is still worth exploring. Recently, it was found that the inhibition of *Dot1l* can induce 2C-like transcription state with collapsed chromocenters and reduced H3K79me3, representing the remodeled pericentromeric heterochromatin. As a result, MERVL loci were released from the heterochromatic environment and become activated (Xu Y. et al., 2022; Yang et al., 2022).

In addition, when nucleoli were disrupted by blocking RNA polymerase I activity or preventing nucleolar phase separation, *Dux* loci can dissociate from the nucleolar surface and get activated, promoting the transition to 2CLCs (Xie et al., 2022). In mouse embryos, rRNA biogenesis and nucleolus defect prevented nucleolar maturation, resulting in developmental arrest at the 2C–4C stage (Zhu et al., 2021a; Xie et al., 2022).

The topology in the genome is mediated by CTCF (CCCTC-binding factor) and cohesin (Merkenschlager and Nora, 2016). CTCF can act as a transcriptional activator through interaction with RNA PolII (Chernukhin et al., 2007), and it can also act as a transcription repressor when functioning together with the histone deacetylase complex (Lutz et al., 2000). Depletion of CTCF or cohesion (Zhu et al., 2021b) facilitated ESCs to transition into 2CLCs (Song et al., 2022) with chromatin relaxation and activation of *Dux* loci (Olbrich et al., 2021). The transition from totipotency to pluripotency during embryonic development was also characterized by the progressive accumulation of CTCF and maturation of TADs (Du et al., 2017; Ke et al., 2017; Chen et al., 2019). CTCF also modulated the 3D genome to enable pluripotency reprogramming by acting as an insulator along with imitation switch chromatin remodeler *Smarca5* (Song et al., 2022). However, another study showed that *Smarca5* positively regulates 2C-like transcription in a *Dppa2*-dependent manner (Alda-Catalinas et al., 2020). Thus, it was possible that *Smarca5* and CTCF may potentially act together or independently to exert cell state-specific transcription functions.

In general, 3D genome is closely related to the chromatin organization and also influences the rewiring of the transcription network.

## 4 Cell cycle and DNA damage response in totipotency

Cell fate decision processes, such as differentiation and reprogramming, are typically coupled with cell cycle progression (Gonzales et al., 2015; Gruenheit et al., 2018). Totipotent stem cells potentially possess distinct cell cycle patterns compared with cells from other developmental stages. For instance, 2CLCs, and also 2C embryo cells, have been shown to slow down the DNA replication fork speed (Nakatani et al., 2022). 2CLCs were found to emerge along with slow DNA replication, and genes highly expressed in 2CLCs had distinct replication timing (RT), mainly during the early S-phase. The slow replication fork speed enabled the remodeling of replication timing in MERVL-enriched specific genomic regions toward the early S-phase, which led to their activation (Nakatani et al., 2022). In general, slowing down the replication fork speed induced 2CLCs and improved the reprogramming efficiency of somatic cell nuclear transfer (Nakatani et al., 2022).

In addition, the totipotency state is usually associated with prolonged G2/M phases (Eckersley-Maslin et al., 2016; Atashpaz et al., 2020; Zhu et al., 2021a). In *Dux* over-expression-induced 2CLCs, those with higher MERVL expression (2C-IN) exhibited a prolonged G1 phase, while cells with lower MERVL expression (2C-OUT) exhibited a prolonged G2/M phase (Zhu et al., 2021a). However, whether and how specific cell cycle-related pathways and proteins play a role during the 2CLC transition from ESCs remains elusive. The influence of the cell cycle on the 2C-like state may be partly due to the regulation of the nucleolar structure. During the cell cycle progression, dynamic remodeling occurs in the nucleolus (Probst et al., 2009; Padeken and Heun, 2014; Nagano et al., 2017; Zhang H. et al., 2019). Immature nucleolus structure and reduced rRNA synthesis were found in 2CLCs and 2C embryos (Xie et al., 2022). Interestingly, it was demonstrated that LIN28 could interact with the TRIM28/NCL complex to mediate the repression of *Dux* in mESCs and pre-implantation embryos (Percharde et al., 2018; Sun et al., 2022). When rRNA biogenesis was inhibited, the TRIM28/NCL/LIN28 complex dissociated from peri-nucleolar heterochromatin (PNH), which led to *Dux* loci releasing from PNH and activation of MERVL and 2C genes (Yu et al., 2021). Similar changes in nucleoli were also observed when mESCs were arrested by DNA synthesis inhibitor cytarabine CYT (Zhu et al., 2021a). CYT triggered 2C gene activation independent of the ATR-CHK1 and the p53 pathways (Zhu et al., 2021a).

Cell cycle progression and DNA synthesis also potentiate the generation of DNA damage. Critically, multiple sources contribute to endogenous DNA damage in early embryo development (Ziegler-Birling et al., 2009; Wossidlo et al., 2010; Srinivasan et al., 2020). Similarly, in *Dux*-induced 2CLCs, researchers observed increased DNA damage and cell death (Guo et al., 2019; Olbrich et al., 2021). DNA damage triggered the activation of ATM/ATR and CHKs, which activated *Grsf1* (G-rich sequence factor 1) to stabilize *Du*x mRNA and induce 2C-like states (Atashpaz et al., 2020). Moreover, *p53*, the downstream effector of DDR, was rapidly activated after fertilization and promoted *Dux* function and ZGA gene expression. Importantly, *p53* expression can rescue *Dux* activation in *Dppa2/4* double knock-out mESCs (Grow et al., 2021). This suggests that the DDR/P53 pathway can participate in the timely activation of totipotent genes.

Although DNA damage and CHK1 activation induced 2CLCs (Atashpaz et al., 2020; Grow et al., 2021), no increase in γH2AX was observed in 2CLCs. Furthermore, depletion of checkpoint proteins did not affect the generation of 2CLCs, leaving the necessity of the DDR pathway in totipotency to be elucidated (Nakatani et al., 2022). In mammalian pre-implantation embryos, DNA replication stress predisposed gene-poor regions to chromatin instability, which impaired developmental potential of cells (Palmerola et al., 2022). The DNA damage resulted in reduced replication fork speed (Tercero and Diffley, 2001). A possible cause of replication fork stalling is DNA demethylation (Guo et al., 2014). Also, γH2AX foci were found in regions with active DNA demethylation in mouse pre-implantation embryos (Wossidlo et al., 2010).

Overall, totipotent-like cells and totipotent embryos potentially possess distinct cell cycle patterns compared with other cells. Meanwhile, DNA replication stress is abundant during mammalian pre-implantation embryos and can activate the 2CLC network (Atashpaz et al., 2020; Grow et al., 2021). In the future, the mechanism of how DNA damage occurs in early embryos and how cell cycle affects embryonic development potential will need to be further elucidated.

## 5 RNA metabolism and totipotency regulation

RNA metabolism, including RNA synthesis, modification, and degradation, can also contribute to gene expression dynamics (Qiu et al., 2020). RNA splicing can regulate mRNA stability and plays an important role in vertebrate development. Interestingly, screening of factors promoting the 2C-like state revealed a large number of candidates belonging to spliceosome and RNA splicing pathways (Rodriguez-Terrones et al., 2018). Recently, *Zrsr1* and *Zrsr*2, minor splicing factors, were found to be essential for ZGA and the transition to 2CLCs from mESCs. *Zrsr1/2* double knock-out embryos arrested at 2–4C stages and RNA-seq revealed remarkable upregulation of RNAs of early embryo development. This was linked to a blockade of maternal RNA degradation (Gómez-Redondo et al., 2020). Remarkably, a broad-spectrum spliceosome inhibitor, plaB, can reverse mESCs into a totipotent transcription network and promote the 2C-like state conversion (Shen et al., 2021). Apart from splicing, the nonsense-mediated mRNA decay (NMD) pathway was also linked to 2C and totipotency states. It was identified to degrade *Dux* RNA upon entry into the 2C state (Fu et al., 2020a). However, as a general spectrum RNA quality surveillance pathway, how NMD specifically facilitate 2C-related RNA degradation during early development is still unknown. In addition, using an unbiased genome-scale CRISPR knock-out screen in mESCs, m6A modification-related genes, such as *Mettl3/14*, were found to repress the RNA abundance of IAPs and ERVK elements, thus promoting the 2C-like transition from mESCs (Chelmicki et al., 2021). With increasing evidence demonstrating the effect of RNA metabolism on the totipotency transcription network and totipotency reprogramming, still more effects are needed to elucidate the molecular bases of RNA metabolism in the context of early development and totipotency.

## 6 Regulation of the signaling pathway

The signaling pathway is an essential way of signal transmission between cells and with the external environment. Key signaling pathways are of pressing importance during embryonic development. The expanded developmental potential of EPSCs was thought to be mediated by high BMP4 and low activin/nodal signaling activity. In a similar manner, human blastoid formation was improved by triple inhibition of the Hippo, TGF-β, and ERK pathways with chemical inhibitors (Malik and Wang, 2022). The active Akt signaling pathway supports self-renewal of ESCs; in contrast, Akt-specific inhibitors API-2 and MK2206 could induce 2C arrest and significantly downregulated the mRNA of MERVL and eIF-1A, implying p-Ser473-Akt may be a potential player in the totipotency transcription in 2C mouse embryos (Chen et al., 2016).

Retinoic acid (RA), a metabolite of vitamin A, displays important roles during embryonic development. Several studies reported that RA drove a NELFA-mediated 2C-like state under S/LIF culture conditions (Sharova et al., 2016; Tagliaferri et al., 2020; Iturbide, 2021; Wang et al., 2021) through a coordinated expression of *Dux* and *Duxbl1* and the activation of *Prame* family member *Gm12794c* (Napolitano et al., 2020). Not only transcriptional and developmental potentials but also DNA methylation, histone modification, glycolysis, and protein synthesis exhibit similarities to 2C embryos in RA-induced 2C-like cells (Wang et al., 2021). However, RA-treated cells gradually died or differentiated, and they failed to maintain a stable totipotent state (Hu et al., 2022). In the pluripotent state, RA counteracted the differentiation and maintains pluripotency, through the activation of WNT signaling (Wang et al., 2008), *Nanog* expression (Chen et al., 2007), and phosphoinositide 3-kinase (PI3K) activation (Chen and Khillan, 2010). Chemical manipulation of signaling pathways could further improve the totipotency reprogramming efficiency and may lead to the conversion toward a more functional totipotency state *in vitro* (Malik and Wang, 2022).

## 7 Understanding the totipotency state from integrated angles

The establishment of a stable *in vitro* totipotent stem cell culture system has been a long-term goal of the stem cell and early developmental biology field. Currently, various *in vitro* culture systems have been identified or established, including 2CLCs (Macfarlan et al., 2012), EPSCs (Yang Y. et al., 2017; Yang et al., 2017 Y.), TBLCs (Shen et al., 2021), TLSCs (Yang et al., 2022), ciTotiSCs (Hu et al., 2022), and TPS cells (Xu Y. et al., 2022). Cells from these models mimicked totipotent transcriptome to different extents and can give rise to intra- and extraembryonic lineages under different experimental contexts.

*In vitro* totipotent state is a dynamic state, and the cells induced to reprogram the 2C-like state were found to spontaneously roll back to the original pluripotent state (Macfarlan et al., 2012; Rodriguez-Terrones et al., 2018; Olbrich et al., 2021). It is thought that dynamic alteration in the transcriptome may be the underlying cause. Negative feedback responses were evident in the 2CLC transcription network, which may be associated with the instability of 2CLCs. For instance, *Dppa2* and *Dppa4* formed a negative feedback loop involving *Dux* and *Du*x repressors LINE-1 (De Iaco et al., 2019). Otherwise, ZSCAN4 bound to a CA repeat embedded in the *Dux* upstream of the TSS unit and downregulated *Dux* expression in the presence of double-stranded DNA breaks (Grow et al., 2021). In addition, *Duxbl1* expression and 2CL state were induced by the treatment of RA (Sharova et al., 2016; Tagliaferri et al., 2020; Iturbide, 2021; Wang et al., 2021); however, in the meantime, *Duxbl1* can directly downregulate *Zscan4* and endogenous *Duxbl1* (Tagliaferri et al., 2020). These shreds of evidence indicated negative feedback regulation exists in the 2CLC transcriptional regulatory network and may underlie the instability of *in vitro* totipotency obtained so far. Moreover, early totipotent embryos and totipotent-like cells exhibit low metabolism and DNA damage activation. These properties argue against a self-sustainable totipotency state which can be stably maintained in the long term.

Using transcriptome profiling of the *Dux*-induced 2CLC system, Fu et al. (2020a) found that the entry and exit from the 2C-like state follow distinct transcription pathways. NMD-mediated mRNA decay of *Dux* seems to play a very important role during the 2C exit process. Apart from RNA metabolism, biological processes negatively regulating the 2C state may be involved in the exit from totipotency and embryonic development after 2C. RNA splicing, RNA decay (Fu et al., 2020a; Shen et al., 2021), and rRNA biosynthesis (Grow et al., 2021; Yu et al., 2021) were all shown to exhibit negative regulatory effects on 2CLCs (Figure 1). *Prdm14a*, a pluripotency transcription factor, acted as a barrier in the transition of mESCs to 2CLCs (Nakaki and Saitou, 2014). *Myc* can prevent the transition to the 2C-like state by actively maintaining the pluripotent transcriptome (Fu et al., 2019). *Otx2*, a member of the OTX family highly expressed in mouse blastocysts and ESCs, negatively regulated *Dux*, *Zscan4c*, MERVL, and other ZGA genes in mESCs (Guo et al., 2021). Highly abundant *TRIM66/DAX1* in mESCs served as a negative regulator of the 2C-like state through repressing *Dux* transcription (Zuo et al., 2022). These factors may all be involved in the dissolution of the 2C-like state.

Therefore, the exit from the *in vitro* 2C-like state also mimics the developmental process from 2C to morula and blastocyst stages. Understanding whether 2C embryos also exhibit similar 2C exit mechanisms as *in vitro* 2C-like cells and whether the totipotency state is intrinsically associated with a dynamically wired and unstable transcription state has great implications for us to understand the transcriptional properties in early embryo development.

## 8 Summary and outlook

The establishment of a totipotent-like system *in vitro* greatly helped us chart the map of embryonic development. Given the knowledge about totipotency obtained so far, large gaps still exist in fully understanding the regulatory and functional properties of the totipotency state. Totipotency represents the highest developmental potential in early embryogenesis and thus potentially can contribute toward the widest applications of stem cells such as reproduction, development, and tissue/organ regeneration. It is evident that further understanding of totipotency regulation and optimizing chemically defined culture conditions will further increase the efficiency of totipotency reprogramming and create more functional *in vitro* totipotency models for basic research and regenerative medicine (Malik and Wang, 2022).
